# Glibenclamide attenuates myocardial injury by lipopolysaccharides in streptozotocin-induced diabetic mice

**DOI:** 10.1186/s12933-014-0106-y

**Published:** 2014-07-31

**Authors:** Jian Cai, Shuai Lu, Zheng Yao, Ya-Ping Deng, Ling-Di Zhang, Jia-Wen Yu, Guo-Fei Ren, Fu-Ming Shen, Guo-Jun Jiang

**Affiliations:** Department of Pharmacy, Zhejiang Xiaoshan Hospital, Hangzhou, Zhejiang 311202 China; Department of Pharmacy, Shanghai Tenth People’s Hospital, Tongji University, Shanghai, 200072 China

**Keywords:** Glibenclamide, Lipopolysaccharides, Myocardium injury, Diabetes mellitus, Inflammation

## Abstract

**Background:**

Sepsis is a common disease that continues to increase in incidence in the world. Diseases, such as diabetes mellitus, may make the situation worse. Diabetic patients are at increased risk for common infections. This study was designed to investigate the role of glibenclamide on myocardial injury by lipopolysaccharides (LPS) in streptozotocin induced diabetic mice (STZ-mice).

**Methods:**

LPS was used to induce endotoxemia in STZ-mice. Heart rate and mean arterial pressure were measured by MPA-HBBS. Serum epinephrine level was measured by enzyme-linked immunosorbent assays (ELISA). Myocardial injury was examined by light and transmission electron microscope and TUNEL staining. Macrophage infiltration was measured by immunohistochemistry. Interleukin-1β (IL-1β) and tumor necrosis factor-α (TNF-α) levels in myocardial tissue and serum in STZ-mice, and in conditional medium of primary cultured peritoneal macrophages were determined by ELISA. Nalp3 and Caspase-1 protein levels were measured by Western blotting analysis.

**Results:**

STZ administration decreased body weight and increased blood glucose in C57BL/6 mice. LPS injection caused decreases of heart rate and mean arterial pressure, and elevated serum epinephrine level in C57BL/6 mice. Compared with control mice without STZ treatment, LPS induced more severe myocardial injury and macrophage infiltration in STZ-mice, which was attenuated by pretreatment of glibenclamide. LPS stimulation enhanced the levels of IL-1β and TNF-α in both cardiac tissue and serum. Glibenclamide pretreatment significantly inhibited the serum levels of pro-inflammatory cytokines. Either high glucose or LPS increased the levels of IL-1β and TNF-α in the conditional medium of peritoneal macrophages. Glibenclamide treatment suppressed the increase of IL-1β level induced by high glucose and LPS. Furthermore, Nalp3 and Caspase-1 levels were markedly increased by high glucose plus LPS, and both proteins were significantly inhibited by glibenclamide treatment.

**Conclusions:**

We conclude that glibenclamide could attenuate myocardial injury induced by LPS challenge in STZ-mice, which was possibly related to inhibiting inflammation through Nalp3 inflammasomes.

**Electronic supplementary material:**

The online version of this article (doi:10.1186/s12933-014-0106-y) contains supplementary material, which is available to authorized users.

## Introduction

Septic shock induced by bacteremia is one of the leading causes of death in critical patients. The mortality of septic shock ranges from 37% to 47% [1]. Activation of inflammatory factors in septic shock always occurs as a simultaneous immune response program initiated early in the course of the disease. Such as endotoxemia, this occurs frequently in septic shock, cause to hemorrhages, necrosis of the kidneys, and myocardial dysfunction. To the final stage, the progressive systemic organ failures may be developed due to interaction between the severe infection and the hyperactive inflammatory response during septic shock [2].

Lipopolysaccharide (LPS) is considered the principal cause responsible for the heart failure in sepsis shock. LPS may trigger acute and chronic inflammation, leading to immune cell activation and cytokine release [3]. In endotoxemia, hyperactivation of the immune response leads to the excessive production of various pro-inflammatory cytokines (IL-1β and TNF-α) and cellular injury [4], which also can result in a systemic inflammatory response and eventually lead to multiple organ failure and death. However, the precise mechanisms responsible for myocardial dysfunction in the setting of endotoxemia are not fully elucidated [5].

Diabetes mellitus is a group of metabolic disorders characterized by hyperglycaemia resulting from defects in insulin secretion, insulin action or both [6]. With a disease rate of 8.3% and cost of $174 billion, there is no debate that diabetes mellitus is a highly prevalent and costly lifelong disease [7]. It was well-established that all patients with diabetes mellitus were at increased risk for bacterial infections. Moreover, diabetes mellitus was associated with a poorer prognosis among the patients with bacteremia [8]. Therefore, further studies are warranted to manifest the relation between infection and diabetes mellitus, and to deliver more effective management of infections in diabetic patients [9].

Glibenclamide, an ATP-sensitive potassium channel (K_ATP_) blocker, is the most widely used sulfonylurea drug for the treatment of type 2 diabetes mellitus in the United States [10]. It has been shown that glibenclamide suppressed neutrophil migration and chemotaxis during inflammatory responses via blocking K_ATP_ channel [11]. Previously glibenclamide was reported to be able to reduce shock-induced overproduction of pro-inflammatory cytokines during simulated in vivo endotoxinaemia [12]. Importantly, Mohamed *et al.* reported that glibenclamide prevented activation of the Nalp3 inflammasomes [13]. Nalp3 is an essential component of inflammasomes triggered by pathogen-associated molecular patterns, danger-associated molecular patterns, and crystalline substances [14–17]. Inflammasomes activate Caspase-1 for processing and secretion of the cytokines IL-1β and TNF-α [17]. Inappropriate Nalp3 activity has been incriminated in the pathogenesis of several diseases, including gouty arthritis, Alzheimer’s and silicosis [18–20]. Thus, inhibition of the Nalp3 inflammasomes may offer considerable therapeutic promise in inflammatory-associated disease [13]. With LPS induced endotoxemia in STZ-mice, in the present study, we hypothesized that glibenclamide could attenuate myocardial injury through inhibiting inflammation by preventing activation of Nalp3 inflammasomes.

## Materials and methods

### Animals

Male C57BL/6 mice weighing 20 g, about 7 weeks of age, obtained from the SLAC Laboratory Animal Co., Ltd. (Shanghai, China). The animals were maintained at 23°C ± 2°C under a cycle of 12 h light/12 h darkness with free access to food and water. All the animals used in this study received humane care in compliance with the institutional animal care guidelines and the Guide for Care and Use of Laboratory Animals published by the National Institutes of Health.

### STZ-induced diabetic mice

Animals were intraperitoneally injected with a single dose of STZ (Amresco, USA) at 60 mg/kg body weight, dissolved in 0.1 mM sodium citrate buffer (pH 4.5) [21]. On the fifth day after STZ administration, whole blood was obtained from the mice tail vein and glucose levels were measured using the blood glucose monitoring system (MAJOR, Taiwan). For the present study, hyperglycemia is defined as a blood glucose measurement of 20 mM or higher. Citrate buffer-treated mice were used as a normoglycemic control (blood glucose < 12 mM). The STZ-mice were randomly divided into 3 groups: hyperglycemic mice treated with vehicle and glibenclamide-treated mice (per day, 5 or 20 mg/kg, i.g, ×14 d) [11,22,23].

### Endotoxemia model

The endotoxemia was induced by administration of LPS (15 mg/kg, Escherichia coli 0111:B4, Cat. L2630, lot 028 K4090; Sigma-ALDRICH, USA). Six hours after intraperitoneal LPS injection, heart rate and mean arterial pressure were recorded using MPA-HBBS (Shanghai Alcott Biotech CO., LTD, Shanghai, China), as previously described [24]. Serum epinephrine, IL-1β and TNF-α were measured with commercially available ELISA kits (epinephrine: Cloud-Clone Corp, Lot:L140404193, Wuhan, China; IL-1β and TNF-α: R&D Systems, Minneapolis, MN, USA) [25].

### Morphological analysis

For histopathological observation, the mice were sacrificed at 6 h after LPS injection. Heart tissues were fixed in 10% formalin, embedded in paraffin, sectioned, and then stained with hematoxylin and eosin (H&E) for morphological analysis. Transmission electron microscope studies were performed as previously described [5]. Tissues were fixed with 2.5% glutaraldehyde in 0.1 mol/l cacodylate buffer, pH 7.4, for 1 h at 4°C. After rinsing in cacodylate buffer, tissues were postfixed in 1% cacodylate-buffered osmium tetroxide for 2 h at room temperature, and then dehydrated in a graded series of ethanol, transferred to propylene oxide, embedded in Epon-Araldite. Ultrathin sections (60-to-80-nm thick) were cut with a diamond knife, placed on formvar carbon–coated copper grids (200 mesh), and stained with uranyl acetate and lead citrate. Morphometric analyses were observed with a Hitachi H-800 Transmission Electron Microscope (Hitachi, Japan) [26,27].

### Apoptotic analysis

Terminal deoxynucleotidyl-transferase-mediated dUTP-nick-end labeling (TUNEL) staining was performed on formalin-fixed, paraffin-embedded sections with a commercial kit (Boehringer Mannheim, Mannheim, Germany) according to the instructions. Briefly the sections were first deparaffinized with xylene and ethanol and slides rinsed twice with phosphate buffered saline (PBS), treated 15 min with 20 mg/ml proteinase K (Boehringer Mannheim) in 0.1 mol/L Tris–HCl buffer (pH 7.4), then again rinsed twice with PBS. After adding the total volume (50 μl) of enzyme solution (TdT) to the remaining 450 μl of labeled solution (dUTP) to obtain 500 μl TUNEL reaction mixture, each sample was incubated with 50 μl TUNEL reaction mixture at 37° for 60 min and the slides rinsed three times with PBS. After drying, the sample was incubated with 50 μl converter-peroxidase (POD) at 37°C for 30 min and slides were rinsed three times with PBS. Then, 50 μl diaminobenzidine (DAB) substrate was added and the sample incubated for a further 10 min at 20°C before again rinsing slides three times with PBS. Omission of the TdT enzyme in the TUNEL reaction was used as a negative control and resulted in no staining. Apoptosis was evaluated by computer-assisted image analysis system (LEICA QUIPS, LEICA Imaging Systems LTD, England) and the results were calculated as the number of positive-staining nuclei per 1,000 cells. For these counts, 2,000 cells were randomly selected from each specimen [28].

### Immunohistochemical staining

The infiltration of macrophages was assessed using immunohistochemical assays. Sections (2 μm) were dewaxed, incubated with 3% H_2_O_2_, blocking serum, and thereafter with a polyclonal antibody against CD68 (Wuhan goodbio technology CO., LTD, GB13067-1, China) at 1:500 dilution. The sections were rinsed with TBST and biotinylated secondary antibody against rabbit IgG (KPL, 074–1506) for CD68 in a 1:200 dilution. After rinsing with TBST, the sections were incubated with HRP-conjugated streptavidin solution (Dako). HRP labeling was detected using a peroxide substrate solution with 0.8 mmol/L DAB and 0.01% H_2_O_2_. The sections were counterstained with hematoxylin before being examined under a light microscope. Image Pro Plus 6.0 software was used to transfer the interesting area staining density to an integrated optical density (IOD) which reflected the staining level of the area of interest [29–31].

### Measurement of IL-1β and TNF-α in heart tissue and serum

Mice were anesthetized with an intraperitoneal injection of sodium pentobarbital (100 mg/kg). Blood samples and myocardial tissues were collected immediately. To measure cytokine, approximately 50 mg of cardiac tissue was transferred to a tube, homogenized in 1,000 μl Phosphate Buffer Solution, and then centrifuged at 3,500 rpm for 15 min at 4°C temperature, after which the upper layer was collected for further analysis. Blood samples were centrifuged (1,500 rpm for 10 min) and then supernatants were stored as serum at − 80°C used for analysis. IL-1β and TNF-α were measured by using commercially available enzyme-linked immunosorbent assay (ELISA) kits.

### Measurement of IL-1β and TNF-α in the supernatant of primary peritoneal macrophages

The concentrations of cytokine in the supernatant were measured by ELISA. Briefly, three mice were injected with 1 ml of thioglycollate broth (3% w/v) (Sigma, 70157) days before harvesting peritoneal macrophages by washing the cavities with 4 ml of PBS for two times. Four hours later, 24-well culture plates were washed with 1640 medium to remove lymphocytes. Peritoneal macrophages were resuspended in RPMI 1640 cell culture medium cultivated in plates for 24 h in a 37°C humidified incubator to allow macrophages to attach to the surface [32]. Macrophages were incubated with high glucose (33 mM), or high glucose + LPS (1 μg/ml) [29,33], or high glucose + LPS + glibenclamide (100 μM). Cells incubated without drug treatment served as control. The supernatants were collected 24 h later, and IL-1β and TNF-α were measured.

### Western blot analysis

The lysates (50 μg of protein) were boiled for 10 min and electrophoresed on a 12% SDS-PAGE electrophoresis. The membranes were incubated in PBS containing 5% non-fat dry milk for 4 h at 25°C. The blots were then incubated for 4 h at 25°C with primary antibodies for Nalp3 (1:1,000; Abcam, HK) and Caspase-1 (1:200; SANTA CRUZ, USA), and then incubated with IRDye 800CW-conjugated goat anti-rabbit secondary antibody (1:10,000; Rockland, USA) for 1 hour at 25°C. The infrared fluorescence image was obtained using Odyssey infrared imaging system (Li-Cor Bioscience, Lincoln, NE), and the band were quantified by Image J software (NIH, USA).

### Statistical analysis

Data are presented as mean ± SEM and analyzed by ANOVA followed by the Tukey-Kramer multiple comparisons test when appropriate. *P* < 0.05 was considered statistically significant.

## Results

### Glibenclamide did not modify body weight and blood glucose level in STZ-mice

**Figure 1:**
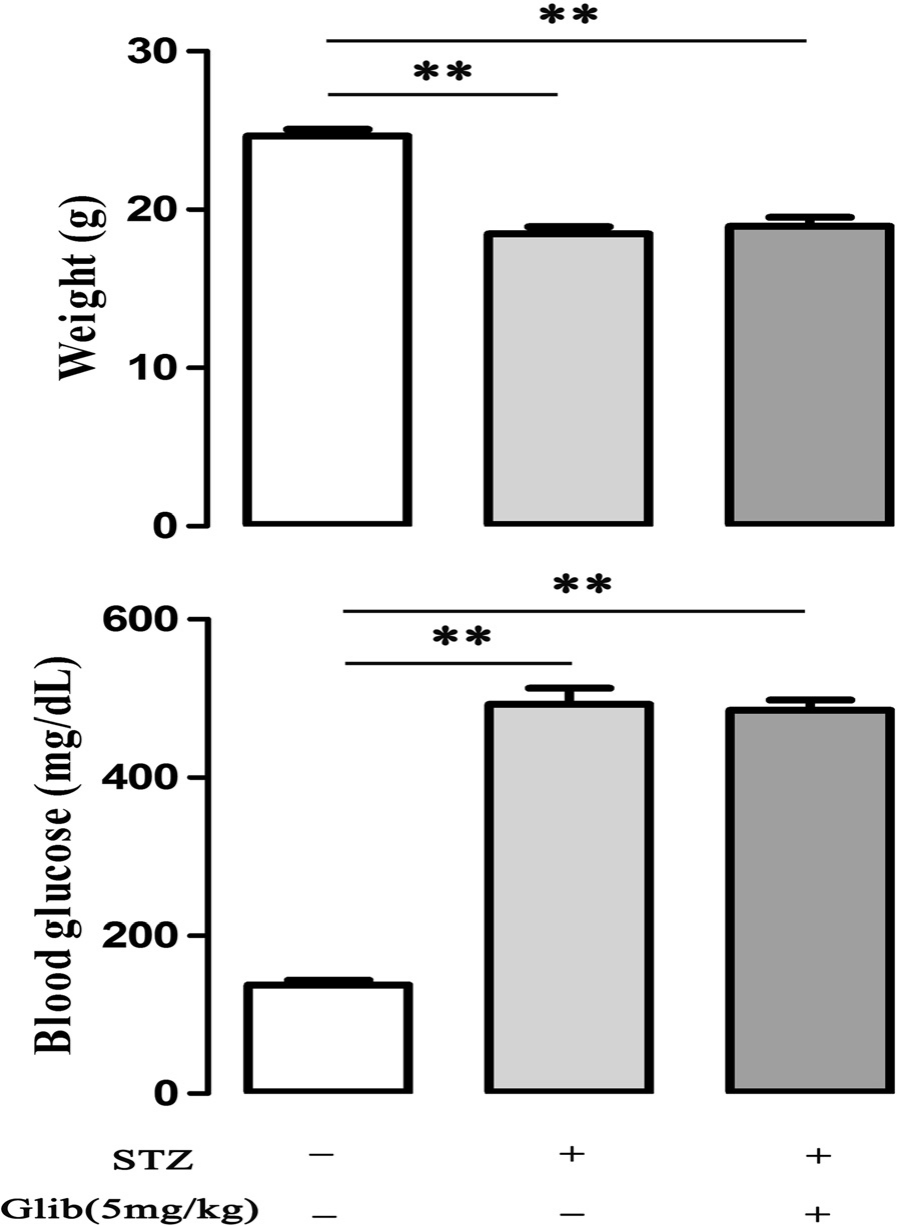
**Glibenclamide did not modify body weight and blood glucose in streptozotocin (STZ) mice.** Compared with control, mice treated with STZ (60 mg/kg, i.p) displayed lower body weight and higher blood glucose, glibenclamide (5 mg/kg, i.g, × 14 d) did not change body weight and blood glucose in diabetic mice. **P < 0.01. Values are means ± SEM (n = 8–10 per group).

As shown in Figure 1, mice were rendered diabetic by the injection of STZ (60 mg/kg, i.p, ×5 d), and then administered by oral gavage glibenclamide (5 mg/kg) for 14 days. Compared with control, STZ-mice exhibited a significant reduction in body weight (18.7 ± 0.4 g *vs* 24.7 ± 0.1 g, *P* < 0.01) and elevation in blood glucose level (492 ± 20.6 mg/dl *vs* 147 ± 3.6 mg/dl, *P* < 0.01). Glibenclamide (5 mg/kg, ×14d) did not change body weight and blood glucose level of STZ-mice (weight: 18.9 ± 0.6 g; blood glucose: 484 ± 13.2 mg/dl, n = 8–10). In addition, oral gavage glibenclamide (5 and 20 mg/kg) for 14 days in normal mice did not change the body weight (23.9 ± 0.6 g vs 23.6 ± 0.2, *P* > 0.05) and blood glucose level (155 ± 8.9 mg/dl vs 159 ± 3.5 mg/dl, *P* > 0.05) (Additional file 1: Figure S1, n = 5).

### Glibenclamide attenuated myocardial injury by LPS in STZ-mice

**Figure 2:**
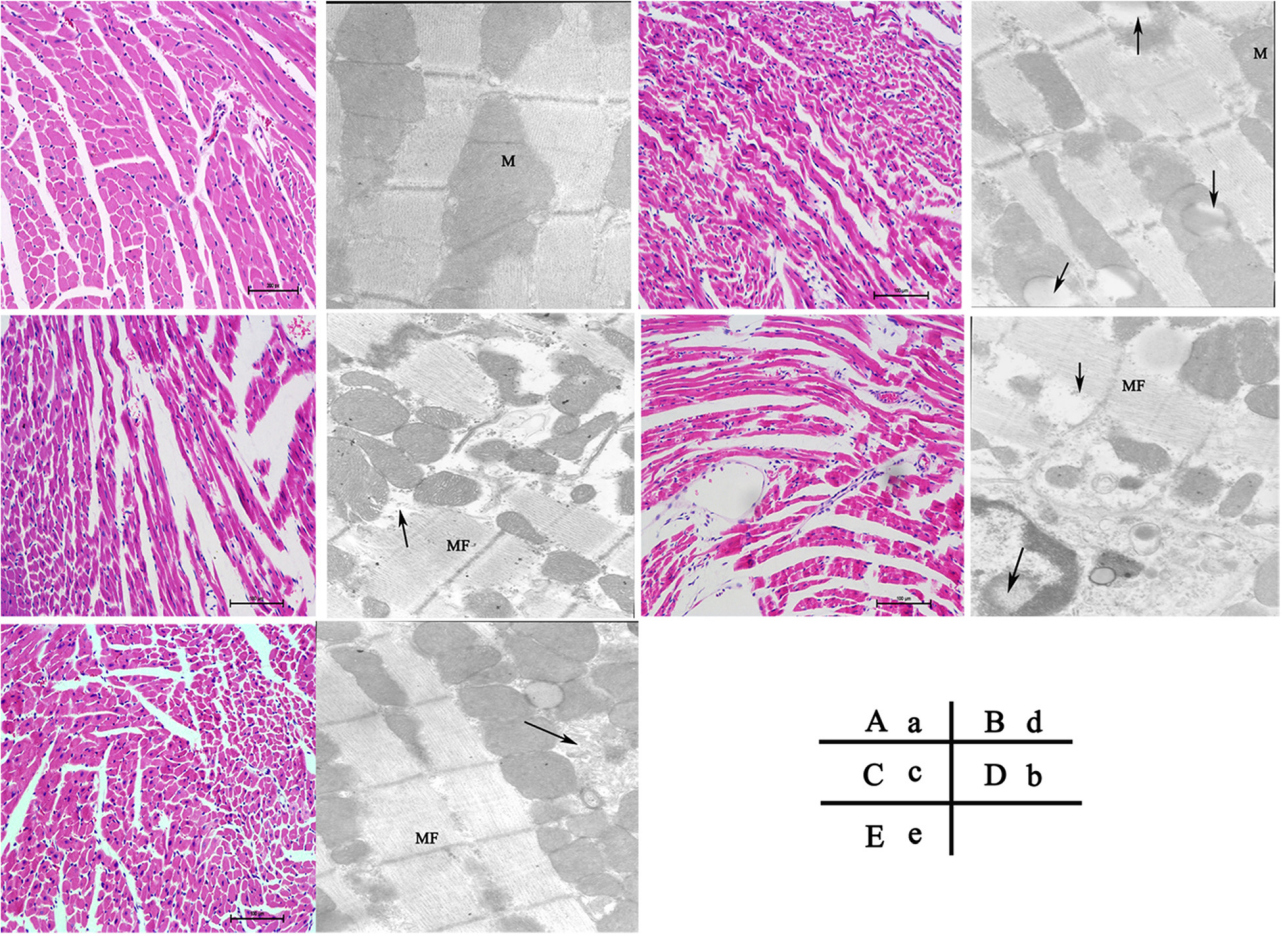
**Morphological analysis of myocardial damage 6 h after LPS administration.** Representative light and transmission electron micrographs of the myocardial tissues indicated that LPS induced more severe myocardial injury in streptozotocin (STZ) diabetic mice, which could be attenuated by glibenclamide (5 mg/kg, i.g, × 14 d) pretreatment. A–E, Hematoxylin-eosin stain ( × 200; n = 3 per group). A: Control, B: STZ, C: LPS, D: S TZ + LPS, E: STZ + LPS + Glib 5 mg/kg. a–e, Transmission electron microscopy ( × 12000; n = 3 per group). a: Control, b: STZ, c: LPS, d: STZ + LPS, e: STZ + LPS + Glib 5 mg/kg. Arrow indicates mitochondria vacuolization and myofilament dissolving. M, mitochondria; MF, myofiber.

Histological analyses were performed 6 h after LPS stimulation. STZ + LPS mice displayed the most serious myocardial injury, including irregular arrangement, degeneration of cardiocytes and rupture of myocardial fibers. Glibenclamide treatment attenuated myocardial injury in STZ + LPS mice. Consistent with the results from light microscopy, transmission electron microscopic analysis showed similar phenomena: the mitochondria of cardiocytes in STZ + LPS mice showed vacuolization and irregular swelling, accompanied by partial myofibers dissolving. Glibenclamide treatment relieved all these changes (Figure 2, n = 3). In addition, in our study, LPS injection caused decreases of heart rate and mean arterial pressure, and elevated serum epinephrine level in mice (Additional file 2: Figure S2, n = 5).

### Glibenclamide attenuated myocardial apoptosis by LPS in STZ-mice

**Figure 3:**
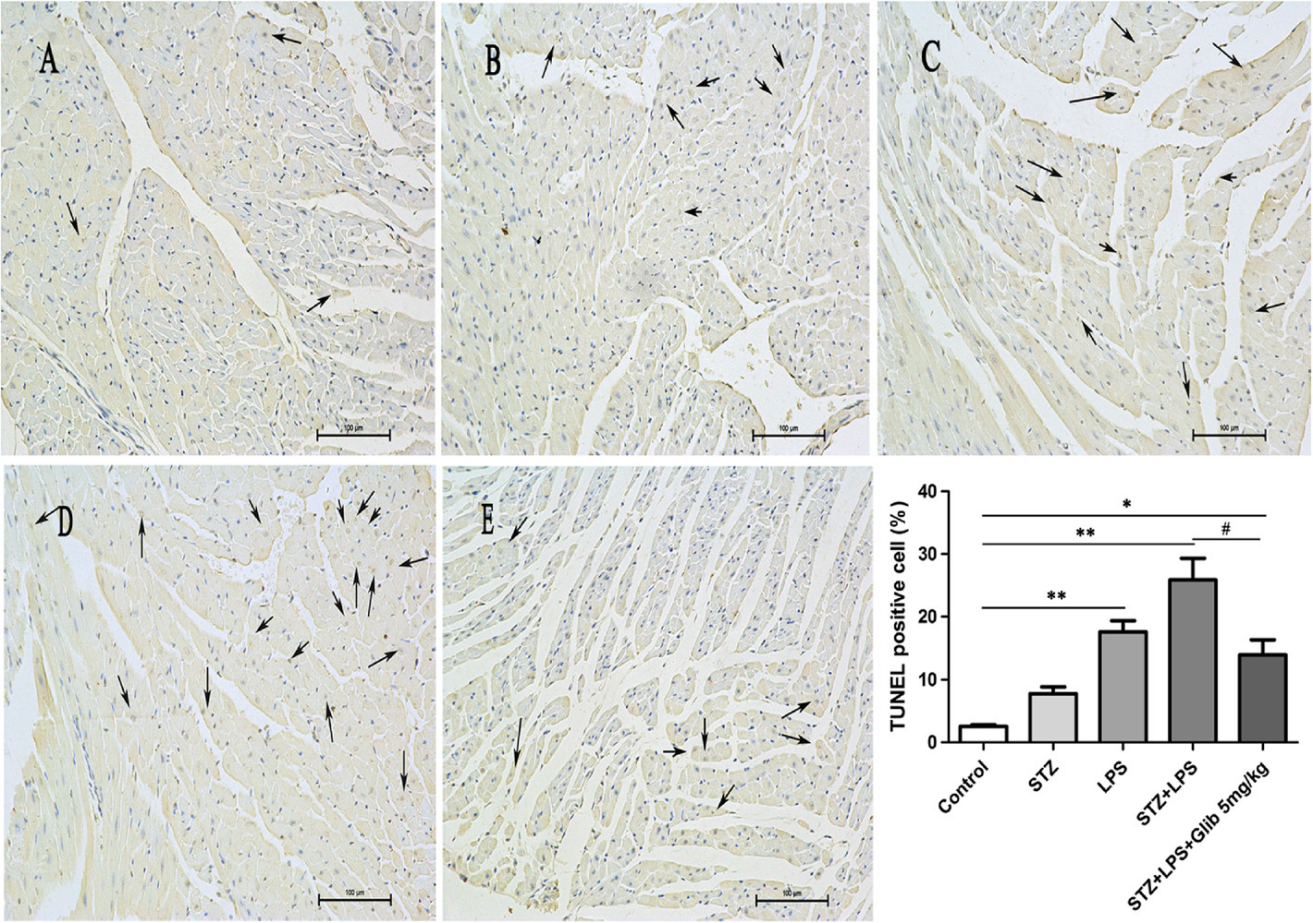
**Glibenclamide inhibited myocardial apoptosis in streptozotocin (STZ) mice after LPS administration.** Representative TUNEL staining micrographs of the myocardial tissues indicated that LPS induced more severe injury in STZ diabetic mice, which could be attenuated by glibenclamide (5 mg/kg, i.g, × 14 d) pretreatment. **A–E**, TUNEL staining ( × 200; n = 3 per group). **A**: Control, **B**: STZ, **C**: LPS, **D**: STZ + LPS, **E**: STZ + LPS + Glib 5 mg/kg. **P < 0.01 *vs* control, ^#^ *P* < 0.05 *vs* STZ + LPS. Values are means ± SEM.

Myocardial apoptosis was examined by TUNEL staining. Apoptotic cells were only occasionally found in the heart of control mice. LPS and STZ + LPS injection caused obvious apoptosis (17.6 ± 1.76% and 25.9 ± 3.5% respectively, *P* < 0.01). Glibenclamide treatment significantly reduced the number of apoptotic cells (14.0 ± 2.3%, *P* < 0.01) (Figure 3, n = 3).

### Glibenclamide decreased macrophage infiltration in the cardiocytes by LPS in STZ-mice

**Figure 4:**
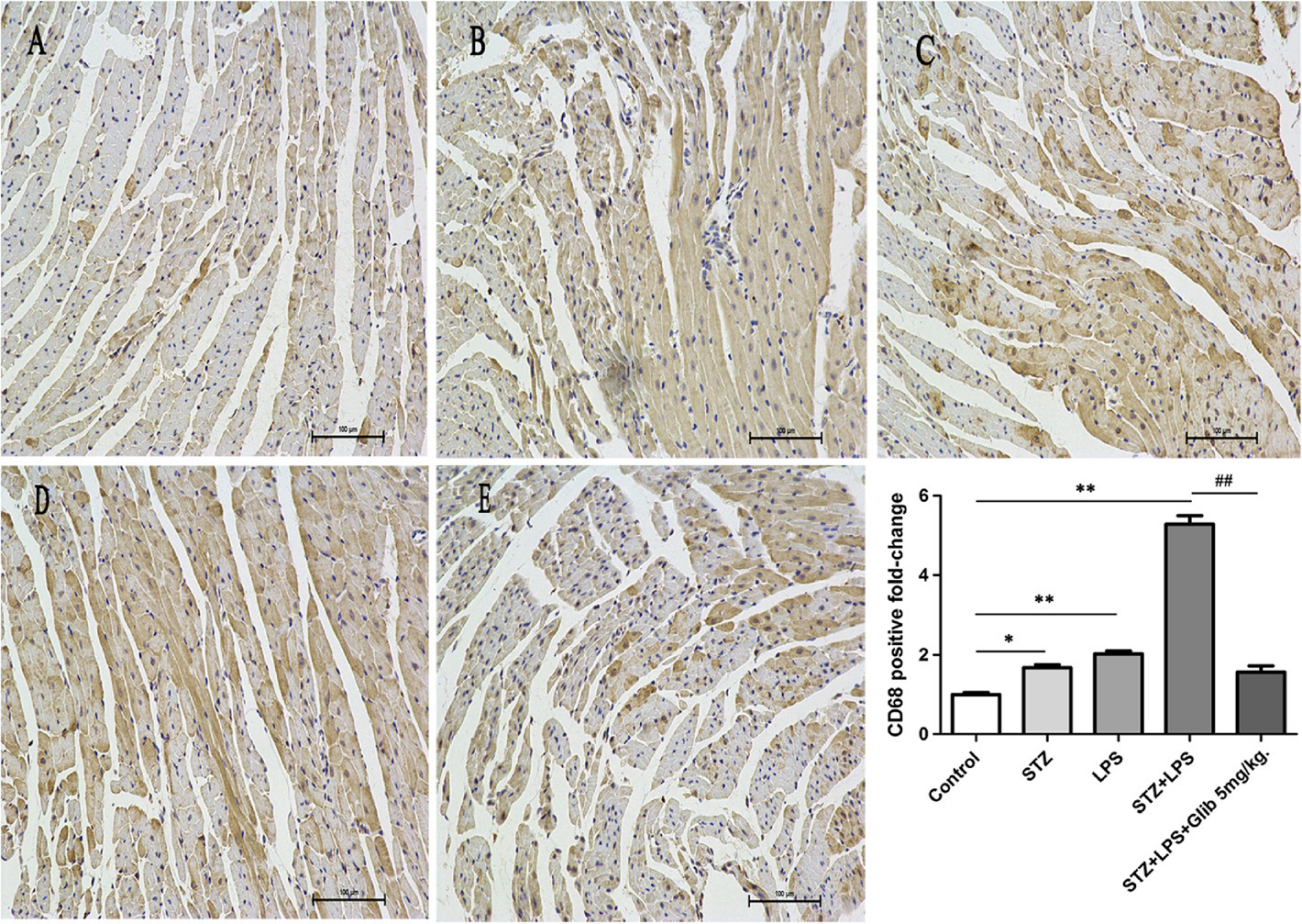
**Glibenclamide inhibited macrophage infiltration in streptozotocin (STZ) mice after LPS administration.** Representative immunohistochemical staining micrographs for CD68 (brown) of the myocardial tissues indicated that LPS induced more macrophage infiltration in STZ diabetic mice, which could be attenuated by glibenclamide (5 mg/kg, i.g, × 14 d) pretreatment. **(A–E)**, Immunohistochemical staining for CD68 ( × 200; n = 3 per group). **A**: Control, **B**: STZ, **C**: LPS, **D**: STZ + LPS, **E**: STZ + LPS + Glib 5 mg/kg. *P < 0.05, **P < 0.01 *vs* control, ^##^ *P* < 0.01 *vs* STZ + LPS. Values are means ± SEM.

Macrophage infiltration was measured by CD68 straining. CD68 positive area rarely appeared in cardiac tissue of in control mice. Both STZ and LPS injection caused infiltration of CD68 positive cells (1.67 ± 0.1 fold, *P* < 0.05; 2.02 ± 0.1 fold, *P* < 0.01). The CD68 positive area in the STZ + LPS group was significantly increased compared with that in the control group (5.28 ± 0.2 fold, *P* < 0.01), whereas glibenclamide treatment inhibited this effect (1.56 ± 0.2 fold vs 5.28 ± 0.2 fold, *P* < 0.01) (Figure 4, n = 3).

### Glibenclamide did not alter the IL-1β and TNF-α levels by LPS in cardiac tissue of STZ- mice

**Figure 5:**
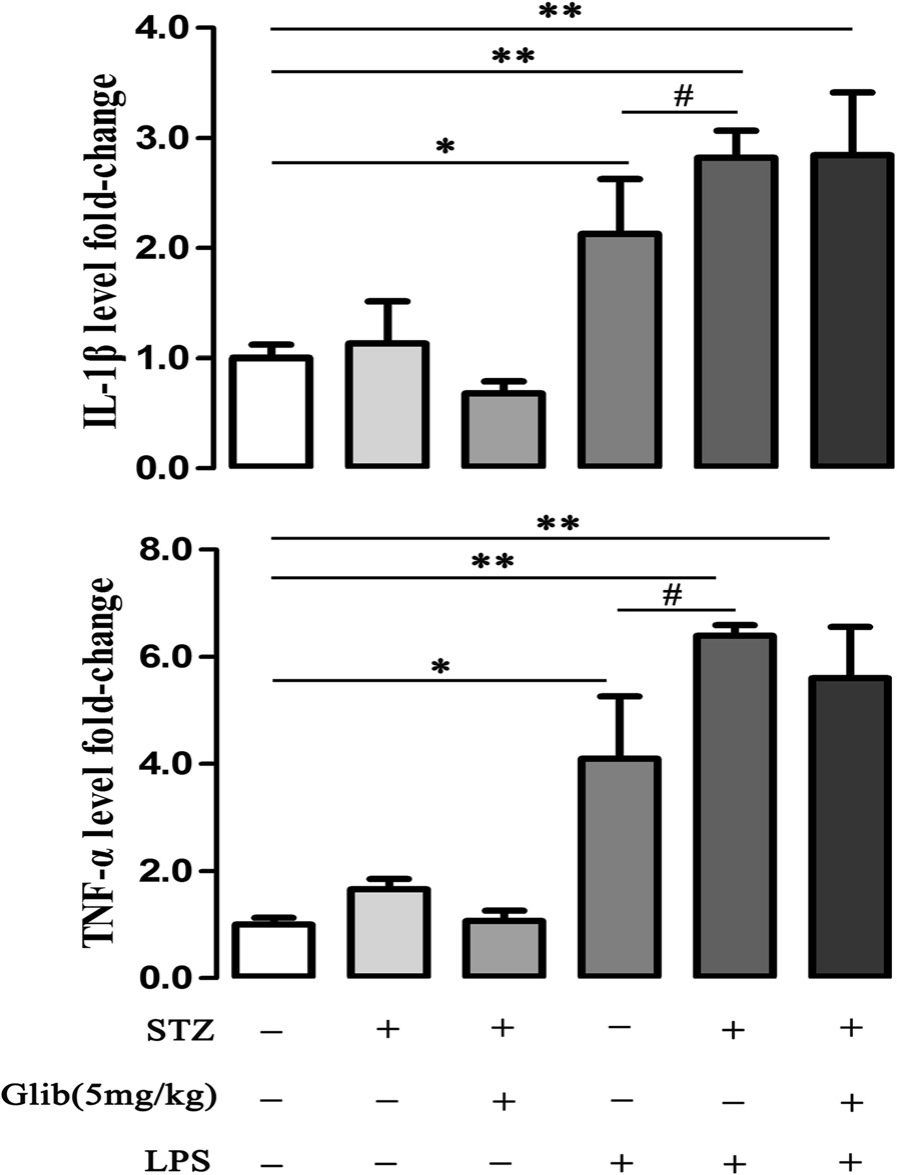
**Glibenclamide did not alter expression of IL-1β** **and TNF-α** **in cardiac tissue by LPS stimulation in streptozotocin (STZ) diabetic mice.** Expression of IL-1β and TNF-α in cardiac tissue were assessed by ELISA 6 h after LPS injection (15 mg/kg, i.p). In non-STZ treated mice, LPS stimulation significantly increased both IL-1β and TNF-α level. In STZ treated mice, LPS stimulation induced greater levels of IL-1β and TNF-α than those in non-STZ treated ones, which were not modified by glibenclamide (5 mg/kg, i.g, × 14 d) pretreatment. *P < 0.05, **P < 0.01 *vs* control, ^#^ *P* < 0.05 *vs* LPS. Values are means ± SEM (n = 8 per group).

LPS injection induced significant increase of IL-1β and TNF-α levels in non-STZ mice (IL-1β: 2.12 ± 0.5 fold, TNF-α: 4.09 ± 1.2 fold, *P* < 0.05). In STZ-mice, LPS induced much higher level of IL-1β and TNF-α (2.83 ± 0.2 fold and 6.39 ± 0.2 fold, *P* < 0.05) compared with those in non-STZ mice (Figure 5, n = 8). Glibenclamide treatment did not change the IL-1β or TNF-α levels in STZ-mice (Figure 5 and Additional file 3: Figure S3, n = 6–8).

### Glibenclamide inhibited serum IL-1β and TNF-α levels by LPS in STZ-mice

**Figure 6:**
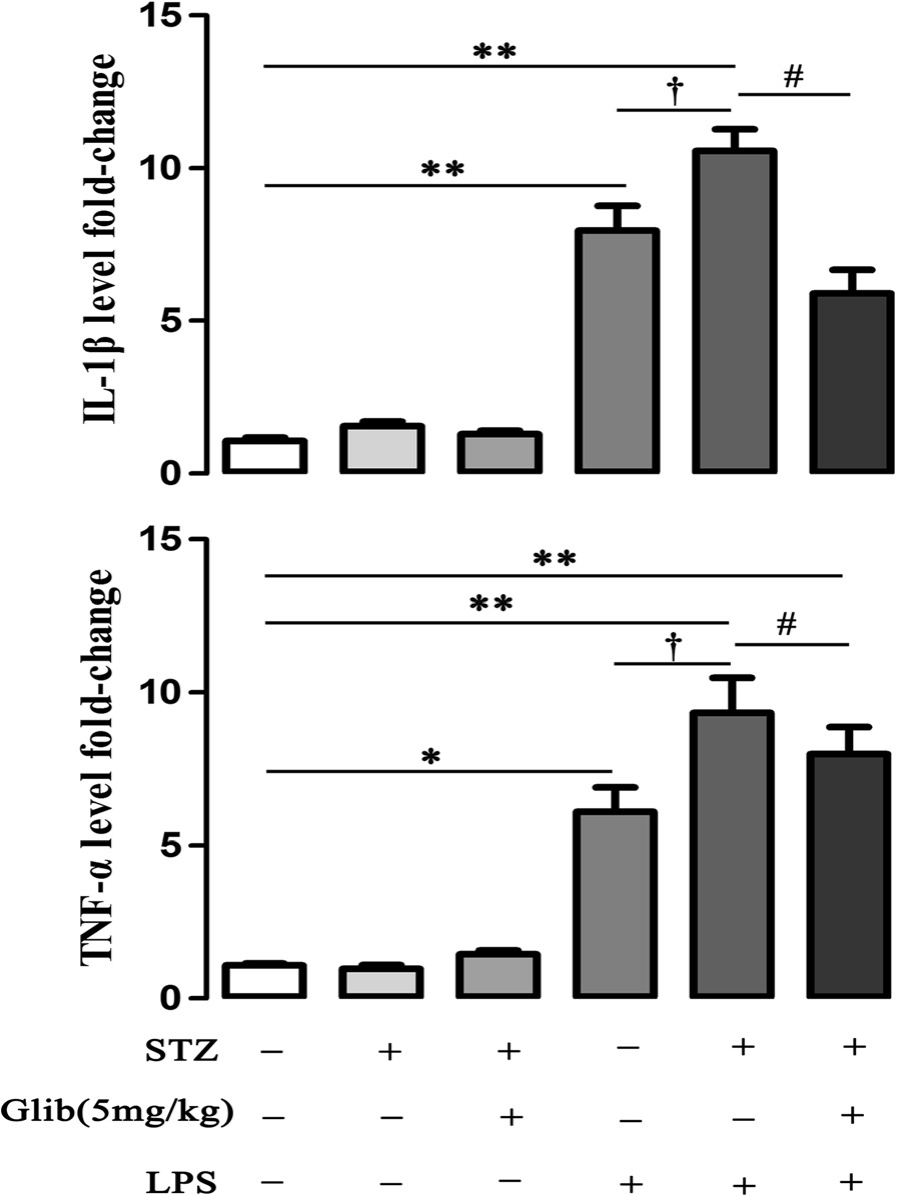
**Glibenclamide inhibited serum IL-1β and TNF-α levels by LPS stimulation in streptozotocin (STZ) diabetic mice.** Serum IL-1β and TNF-α levels were assessed by ELISA 6 h after LPS injection (15 mg/kg, i.p). In non-STZ treated mice, LPS stimulation significantly increased both IL-1β and TNF-α levels. In STZ treated mice, LPS stimulation induced greater levels of IL-1β and TNF-α than those in non-STZ treated ones, which were significantly inhibited by glibenclamide (5 mg/kg, i.g, × 14 d) pretreatment. *P < 0.05, **P < 0.01 *vs* control; ^#^ *P* < 0.05, ^##^ *P* < 0.01 *vs* STZ + LPS, ^†^ *P* < 0.05 *vs* LPS. Values are means ± SEM (n = 8-10 per group).

LPS stimulation induced significant increases of IL-1β (7.96 ± 0.8 fold, *P* < 0.01) and TNF-α (6.09 ± 0.7 fold, *P* < 0.05) levels in non-STZ mice. In STZ-mice, LPS induced greater levels of IL-1β (10.6 ± 0.7 fold), which was significantly decreased by glibenclamide administration (5.89 ± 0.8 fold, *P* < 0.05). After LPS stimulation TNF-α level in STZ-mice reached 9.35 ± 1.1 fold compared to that in non-STZ mice (*P* < 0.05). Similar to IL-1β, glibenclamide treatment significantly inhibited TNF-α expression induced by LPS in STZ-mice (7.97 ± 0.8 fold, *P* < 0.01) (Figure 6, n = 8–10). Compared with LPS + STZ + Glib (5 mg/kg) group, glibenclamide (20 mg/kg) treatment did not change IL-1β or TNF-α level in STZ-mice (IL-1β: 8.23 ± 0.4 fold, TNF-α: 9.61 ± 0.6 fold, Additional file 3: Figure S3, n = 6–8).

### Glibenclamide inhibited IL-1β expression by high glucose and LPS in cultured primary peritoneal macrophages

**Figure 7:**
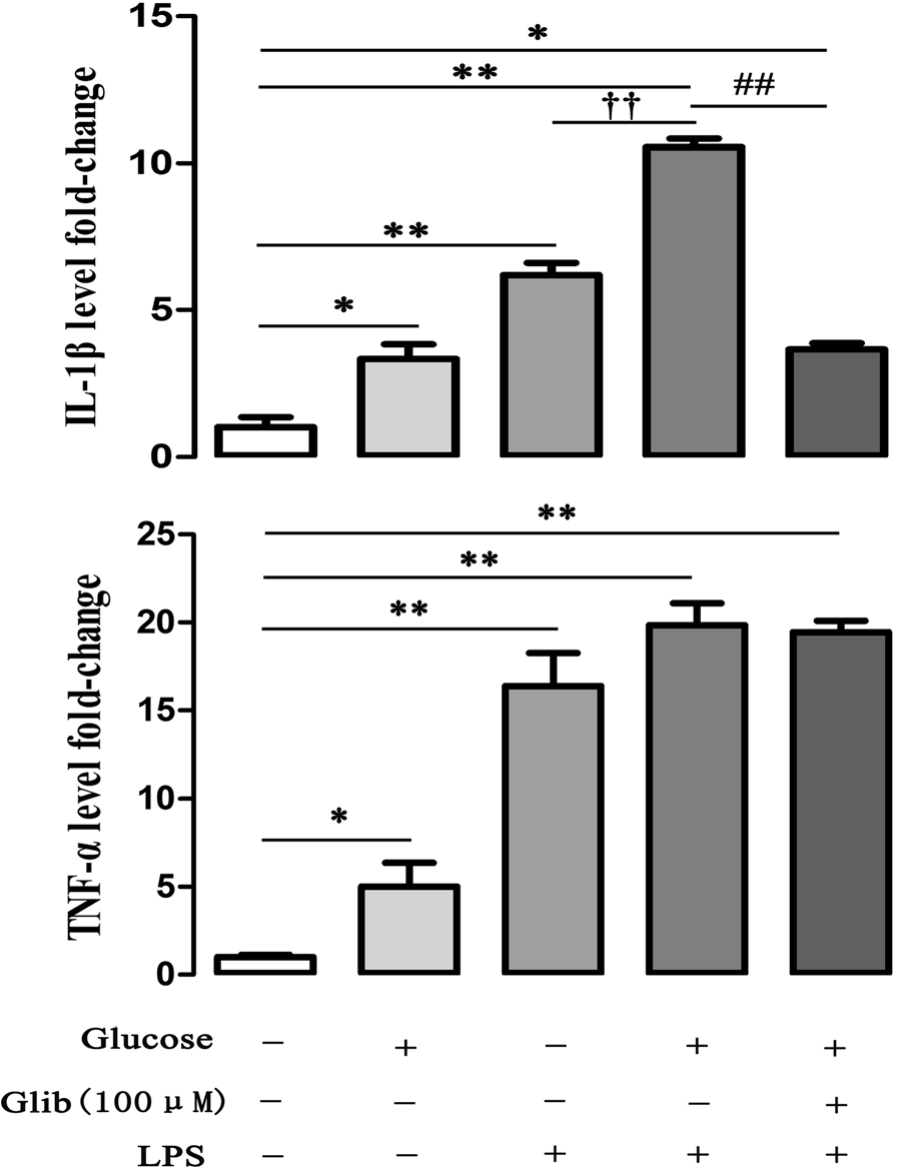
**Glibenclamide inhibited IL-1β expression by high glucose and LPS stimulation in primary peritoneal macrophages.** IL-1β and TNF-α in the supernatants of macrophages were assessed by ELISA. Macrophages were incubated for 24 h with high glucose (33 mM), or high glucose + LPS (1 μg/ml), or high glucose + LPS + glibenclamide (100 μM). Cells incubated without drug treatment served as control. Either high glucose or High glucose + LPS induced significant increase of both IL-1β and TNF-α, glibenclamide treatment only significantly inhibited IL-1β expression by high glucose + LPS stimulation. *P < 0.05, **P < 0.01 *vs* control; ^##^ *P* < 0.01 *vs* Glucose + LPS, ^†^ *P* < 0.05 *vs* LPS. Values are means ± SEM (n = 6 per group).

LPS treatment induced significant increase of IL-1β (6.19 ± 0.4 fold, *P* < 0.01) and TNF-α (16.4 ± 1.9 fold, *P* < 0.01) levels in cultured primary peritoneal macrophages. High glucose alone induced significant increase in both IL-1β (3.33 ± 0.5 fold, *P* < 0.05) and TNF-α (5.01 ± 1.3 fold, *P* < 0.05) level in cultured primary peritoneal macrophages. IL-1β levels were significantly higher (10.5 ± 0.3 fold, *P* < 0.01) in high glucose with LPS treated cultured cells than that was only treated with LPS (6.18 ± 0.4 fold). Glibenclamide pretreatment only significantly decreased IL-1β concentration (3.44 ± 0.2 fold, *P* < 0.01) in high glucose + LPS stimulated cells (Figure 7, n = 6).

### Glibenclamide inhibited Nalp3 and Caspase-1 expression by high glucose and LPS in cultured primary peritoneal macrophages

**Figure 8:**
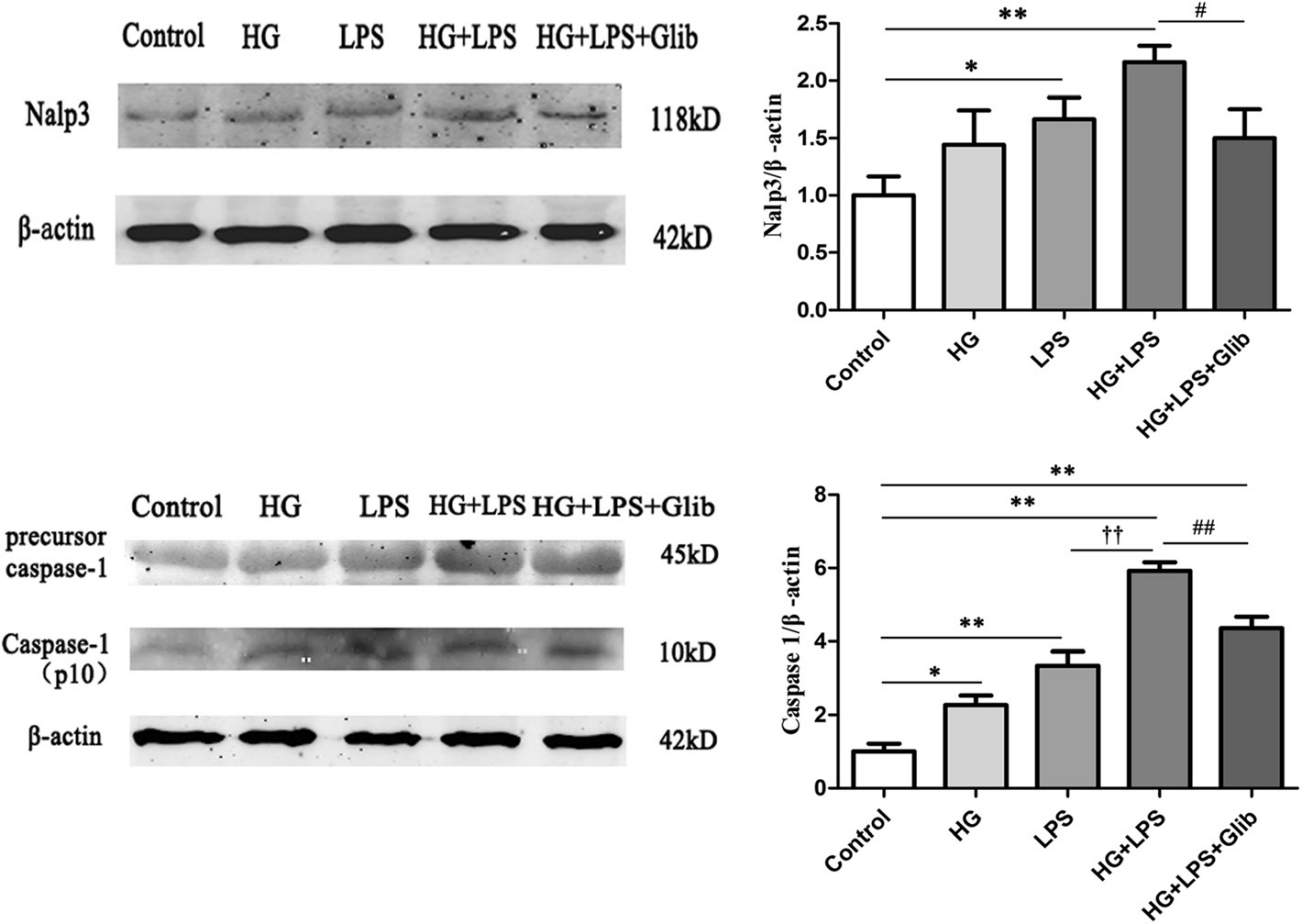
**Glibenclamide inhibited Nalp3 and Caspase-1 expression by high glucose and LPS stimulation in primary peritoneal macrophages.** Nalp3 and Caspase-1 in macrophages were measured by western blotting. Macrophages were incubated for 24 h under high glucose (33 mM), or high glucose + LPS (1 μg/ml), or high glucose + LPS + glibenclamide (100 μM). Cells incubated without drug treatment served as control. High glucose stimulation significantly increased Caspase-1 expression. Nalp3 and Caspase-1 levels were markedly increased by high glucose + LPS challenge. Caspase-1 level was further increased by high glucose + LPS compared with LPS group. Both proteins were significantly inhibited by glibenclamide treatment. *P < 0.05, **P < 0.01 *vs* control; ^#^ *P* < 0.05, ^##^ *P* < 0.01 *vs* Glucose + LPS; ^††^ *P* < 0.01 *vs* LPS. Values are means ± SEM (n = 3 per group).

Cryopyrin/NALP3/NLRP3 is an essential component of inflammasomes triggered by microbial ligands, danger-associated molecular patterns (DAMPs) and crystals. Inflammasomes activate Caspase-1 for processing and secretion of the cytokines IL-1β. Both high glucose and LPS increased Nalp3 expression (1.37 ± 0.1 fold and 1.67 ± 0.2 fold respectively, *P* < 0.05). High glucose + LPS stimulation further increased Nalp3 expression (2.16 ± 0.1 fold, *P* < 0.05). Glibenclamide pretreatment significantly reduced the expression of Nalp3 (1.50 ± 0.3 fold vs 2.16 ± 0.1 fold, *P* < 0.05). Similar changes were observed in Caspase-1 expression (Figure 8, n = 3).

## Discussion

The major findings of this study are as follows: (i) glibenclamide attenuated LPS-induced myocardial injury in STZ-mice; (ii) glibenclamide reduced serum IL-1β and TNF-α induced by LPS in STZ-mice; (iii) glibenclamide inhibited Nalp3 and Caspase-1 expression by LPS + high glucose stimulation in cultured primary peritoneal macrophages. These together suggested that glibenclamide might protect against myocardial injury under inflammation in diabetes.

Sepsis is a common disease with a growing morbidity around the world. Severe sepsis and systemic inflammation are the leading causes of mortality in critically ill patients, resulting from a systemic oxidative-mediated inflammatory response to severe bacterial infection [34]. It is well known that acute infections lead to difficulty in controlling blood glucose and that infection is the most frequently documented cause of ketoacidosis during diabetes mellitus [35]. In this study, the endotoxemic mice displayed hypotension, decreased heart rate, and elevated serum epinephrine level after LPS stimulation, which was consistent with previous reports [24,36,37]. Prevention or management of sepsis remains a barrier to the successful care of many surgical and traumatic patients with diabetes, needing novel therapies urgently [38]. Type 1 diabetes mellitus is a chronic, multifactorial autoimmune disease that involves the progressive destruction of pancreatic β-cells, ultimately resulting in the loss of insulin production and secretion [39]. This response includes the production of cytokines such as IL-1β that orchestrate the recruitment of inflammatory cells to the islets and mediate direct cytotoxic effects on β-cells [40]. TNF-α is considered to be a possible therapeutic target because it was up-regulated in multiple rodent-obesity models and TNF-α blunted insulin signaling in insulin targeting tissues. It is not only exacerbates inflammatory response through acting as a signal amplifier to induce other inflammatory cytokines production, but also contributes to myocardial hypertrophy and fibrosis, leading to left ventricular remodeling and dysfunction [41].

Experimental and clinical studies have suggested that diabetic state causes a specific diabetic cardiomyopathy independent of vascular complications. This cardiomyopathy is characterized by myocyte hypertrophy and fibrosis and may be responsible for the high incidence of cardiac dysfunction and mortality [42]. Ventricles from diabetic patients show accumulation of glycoproteins, collagen, and active fibroblasts [43]. Elevated inflammatory cytokines have been found in circulation and in the hearts of diabetic patients, contributing to heart failure. Cardiac overexpression of TNF-α has been associated with cardiac hypertrophy and fibrosis, as well with left ventricular dysfunction [44]. Moreover, up-regulation of inflammatory cytokines and chemokines by subcutaneous injection of LPS was significantly more rapid and more pronounced in the diabetic mice compared with normal mice [45]. These indicate that cardiac tissue is sensitive to inflammation and inflammatory response is stronger in diabetic condition. We observed significant elevations of IL-1β and TNF-α levels in serum and cardiac tissue upon LPS challenge in mice, suggesting that both of circulating and local inflammatory reaction may cause cardiac injury under diabetic and septic condition. In addition, we found that glibenclamide was able to inhibit the inflammatory cytokines secreted by peritoneal macrophages after LPS treatment. Interestingly, glibenclamide only inhibited the increases of IL-1β but not TNF-α *in vitro*. We considered this specific inhibition on IL-1β level by glibenclamide implicated the involvement of Nalp3, which regulates IL-1β but not TNF-α. There was an inconsistency of TNF-a data in serum and in the supernatant. We think that the situation in vivo is rather complex while the cell model *in vitro* is simple. The serum TNF-α levels might be influenced by many factors. Glibenclamide may lower the serum TNF- α level in a macrophage-independent manner.

K_ATP_ channels are a type of Kir constituted by heteromultimers of two kinds of proteins. Each channel is formed from four pore-forming Kir subunits (Kir 6.1 or Kir 6.2) complexes with four regulatory sulfonylurea receptor proteins (SUR1 in neuronal/pancreatic beta cells or SUR2 in cardiovascular cells). Glibenclamide is a sulfonylurea drug which binds to the SUR1 domain with 10 to 500 fold higher affinity than to the SUR2 domains [6]. Previous study demonstrated that glibenclamide was able to suppress NALP3 activation independently of K_ATP_ [7]. Thus, in this work, we used glibenclamide as an inhibitor of NALP3.

The mechanism of glibenclamide attenuated LPS-induced inflammation is not fully elucidated. Transient receptor potential melastatin 4 (TRPM4), a calcium-activated non-selective cation channel, is functionally expressed in the heart. TRPM4 has been linked to diverse physiological functions, such as protection against Ca^2+^ overload by cell membrane depolarization, modulation of Ca^2+^ oscillations controlling cytokine production in T lymphocytes and mast cells, and dendritic cell migration [46,47]. Grand *et al.* reported that TRPM4 is inhibited by glibenclamide, a modulator of ATP binding cassette proteins (ABC transporters), such as the cystic fibrosis transmembrane conductance regulator (CFTR) [48]. It was also reported that TRPM4 inhibitors 9-phenanthrol and glibenclamide could attenuate LPS-induced endothelial cell death and hypoxia and re-oxygenation-induced early after depolarizations [49]. Glibenclamide is a potent blocker of the ATP-modulated K^+^ channel in insulin secreting cells and a broadly used anti-diabetic drug. Interestingly, recent study showed that glibenclamide could decrease TNF-α and NF-κB activation after subarachnoid hemorrhage [50]. Moreover, glibenclamide reduced LPS-induced release of IL-1β、TNF-α and PAI-2 mRNA in a concentration-dependent manner through reducing the calcium entry by drug-induced depolarization of hypoxic monocytes in an ex vivo model of human endotoxinaemia under hypoxaemic conditions [12]. Koh *et al.* found that glibenclamide directly reduced the secretion of IL-1β by bone-marrow-derived macrophages in a dose-dependent fashion [51]. In the present study, we further demonstrated that glibenclamide could decrease serum IL-1β and TNF-α level induced by LPS in STZ-induced diabetic mice.

Nalp3 is a ‘general sensor’ for danger signals, representing an important caspase-1-containing inflammasomes and is activated by various pathogens [18], and damage-associated molecules and environmental irritants [40]. Activation of Nalp3 leads to oligomerization and recruitment of apoptosis-associated speck-like protein and pro-caspase-1, with auto-cleavage and activation of Caspase-1. Active Caspase-1 cleaves pro-IL-1β to active IL-1β, which, when secreted, can exert direct cytotoxic effects as well as recruit other inflammatory cells. In this work, we found that glibenclamide could inhibit Nalp3 inflammasomes and Caspase-1 induced by LPS + high glucose stimulation in cultured peritoneal macrophages, suggesting that the effects of glibenclamide on prevention of serum IL-1β and TNF-α might be related to inhibiting Nalp3 inflammasomes and Caspase-1. The mechanism how glibenclamide inhibits NALP3 inflammasomes and suppresses IL-1β secretion is an intriguing question. Lamkanfi *et al.* demonstrated that glibenclamide could inhibit the assembly of the NALP3 inflammasomes in response to stimulation with lipopolysaccharide (LPS) and adenosine triphosphate (ATP) [13]. The ability to suppress NALP3 activation of glibenclamide was independent of its inhibitory effect on K_ATP_ [13]. The authors speculated that glibenclamide acts the upstream of NALP3 and the downstream of P2X7 [13]. However, the precise molecular target of glibenclamide for its inhibitory effect on NALP3 inflammasome has yet to be identified.

## Conclusions

In conclusion, our findings indicate that glibenclamide could ameliorate myocardial injury and reducing IL-1β and TNF-α possibly through inhibiting Nalp3 inflammasomes and Caspase-1 signaling under LPS-induced endotoxemia in STZ diabetic mice.

## Additional files

Additional file 1: Figure S1. Glibenclamide did not modify body weight and blood glucose in normal mice. Compared with control, glibenclamide (5 and 20 mg/kg, i.g, ×14 d) administration did not change body weight and blood glucose. NS means no significant difference. Values are means ± SEM (n = 5 per group). Additional file 2: Figure S2. Injection of LPS caused a decrease in heart rate and mean arterial pressure, and elevated serum epinephrine level in mice. Six hours after LPS (15 mg/kg) injection (i.p), heart rate (A) and mean arterial pressure (B) were measured by MPA-HBBS software. Serum epinephrine (C) level was assessed by ELISA. Mice were anesthetized with chloral hydrate (4%), heart rate and mean arterial pressure of endotoxemic mice decreased during LPS stimulation (heart rate and mean arterial pressure had been measured for 10 minutes). Serum epinephrine level was significantly increased in endotoxemic mice compared with control. **P* < 0.05, ***P* < 0.01. Values are means ± SEM (n = 5 per group). Additional file 3: Figure S3. IL-β and TNF-α levels in cardiac tissue and serum had no significant difference between LPS + STZ + Glibenclamide (5 mg/kg) group and LPS + STZ + Glibenclamide (20 mg/kg) group. IL-1β and TNF-α levels in cardiac tissue and serum were assessed by ELISA 6 h after LPS injection (15 mg/kg, i.p). A: Cardiac tissue, B: Serum. NS means no significant difference. Values are means ± SEM (n = 6-8 per group).

## References

[1] Dombrovskiy VY, Martin AA, Sunderram J, Paz HL (2007). Rapid increase in hospitalization and mortality rates for severe sepsis in the United States: a trend analysis from 1993 to 2003. Crit Care Med.

[2] Jackson SK, Abate W, Parton J, Jones S, Harwood JL (2008). Lysophospholipid metabolism facilitates Toll-like receptor 4 membrane translocation to regulate the inflammatory response. J Leukoc Biol.

[3] Lassenius MI, Pietilainen KH, Kaartinen K, Pussinen PJ, Syrjanen J, Forsblom C, Porsti I, Rissanen A, Kaprio J, Mustonen J, Groop PH, Lehto M (2011). Bacterial endotoxin activity in human serum is associated with dyslipidemia, insulin resistance, obesity, and chronic inflammation. Diabetes Care.

[4] Dauphinee SM, Karsan A (2006). Lipopolysaccharide signaling in endothelial cells. Lab Invest.

[5] Yang ZW, Chen JK, Ni M, Zhao T, Deng YP, Tao X, Jiang GJ, Shen FM (2013). Role of Kir6.2 subunits of ATP-sensitive potassium channels in endotoxemia-induced cardiac dysfunction. Cardiovasc Diabetol.

[6] American Diabetes Association (2006). Diagnosis and classification of diabetes mellitus. Diabetes Care.

[7] Grant RW, Dixit VD (2013). Mechanisms of disease: inflammasome activation and the development of type 2 diabetes. Front Immunol.

[8] Thomsen RW, Hundborg HH, Lervang HH, Johnsen SP, Schonheyder HC, Sorensen HT (2005). Diabetes mellitus as a risk and prognostic factor for community-acquired bacteremia due to enterobacteria: a 10-year, population-based study among adults. Clin Infect Dis.

[9] Muller LM, Gorter KJ, Hak E, Goudzwaard WL, Schellevis FG, Hoepelman AI, Rutten GE (2005). Increased risk of common infections in patients with type 1 and type 2 diabetes mellitus. Clin Infect Dis.

[10] Riddle MC (2003). Editorial: sulfonylureas differ in effects on ischemic preconditioning**--**is it time to retire glyburide?. J Clin Endocrinol Metab.

[11] Pompermayer K, Amaral FA, Fagundes CT, Vieira AT, Cunha FQ, Teixeira MM, Souza DG (2007). Effects of the treatment with glibenclamide, an ATP-sensitive potassium channel blocker, on intestinal ischemia and reperfusion injury. Eur J Pharmacol.

[12] Schmid D, Svoboda M, Sorgner A, Moravcevic I, Thalhammer T, Chiba P, Moslinger T (2011). Glibenclamide reduces proinflammatory cytokines in an ex vivo model of human endotoxinaemia under hypoxaemic conditions. Life Sci.

[13] Lamkanfi M, Mueller JL, Vitari AC, Misaghi S, Fedorova A, Deshayes K, Lee WP, Hoffman HM, Dixit VM (2009). Glyburide inhibits the Cryopyrin/Nalp3 inflammasome. J Cell Biol.

[14] Kanneganti TD, Ozoren N, Body-Malapel M, Amer A, Park JH, Franchi L, Whitfield J, Barchet W, Colonna M, Vandenabeele P, Bertin J, Coyle A, Grant EP, Akira S, Núñez G (2006). Bacterial RNA and small antiviral compounds activate caspase-1 through cryopyrin/Nalp3. Nature.

[15] Kanneganti TD, Lamkanfi M, Kim YG, Chen G, Park JH, Franchi L, Vandenabeele P, Nunez G (2007). Pannexin-1-mediated recognition of bacterial molecules activates the cryopyrin inflammasome independent of Toll-like receptor signaling. Immunity.

[16] Mariathasan S, Weiss DS, Newton K, McBride J, O'Rourke K, Roose-Girma M, Lee WP, Weinrauch Y, Monack DM, Dixit VM (2006). Cryopyrin activates the inflammasome in response to toxins and ATP. Nature.

[17] Lamkanfi M, Dixit VM (2009). Inflammasomes: guardians of cytosolic sanctity. Immunol Rev.

[18] Martinon F, Petrilli V, Mayor A, Tardivel A, Tschopp J (2006). Gout-associated uric acid crystals activate the NALP3 inflammasome. Nature.

[19] Cassel SL, Eisenbarth SC, Iyer SS, Sadler JJ, Colegio OR, Tephly LA, Carter AB, Rothman PB, Flavell RA, Sutterwala FS (2008). The Nalp3 inflammasome is essential for the development of silicosis. Proc Natl Acad Sci U S A.

[20] Dostert C, Petrilli V, Van Bruggen R, Steele C, Mossman BT, Tschopp J (2008). Innate immune activation through Nalp3 inflammasome sensing of asbestos and silica. Science.

[21] Parihar MS, Chaudhary M, Shetty R, Hemnani T (2004). Susceptibility of hippocampus and cerebral cortex to oxidative damage in streptozotocin treated mice: prevention by extracts of Withania somnifera and Aloe vera. J Clin Neurosci.

[22] Mutalik S, Chetana M, Sulochana B, Devi PU, Udupa N (2005). Effect of Dianex, a herbal formulation on experimentally induced diabetes mellitus. Phytother Res.

[23] Ahmadi S, Ebrahimi SS, Oryan S, Rafieenia F (2012). Blockades of ATP-sensitive potassium channels and L-type calcium channels improve analgesic effect of morphine in alloxan-induced diabetic mice. Pathophysiology.

[24] Meziani F, Kremer H, Tesse A, Baron-Menguy C, Mathien C, Mostefai HA, Carusio N, Schneider F, Asfar P, Andriantsitohaina R (2007). Human serum albumin improves arterial dysfunction during early resuscitation in mouse endotoxic model via reduced oxidative and nitrosative stresses. Am J Pathol.

[25] Carmody RJ, Ruan Q, Palmer S, Hilliard B, Chen YH (2007). Negative regulation of toll-like receptor signaling by NF-kappaB p50 ubiquitination blockade. Science.

[26] Cheng L, Ding G, Qin Q, Huang Y, Lewis W, He N, Evans RM, Schneider MD, Brako FA, Xiao Y, Chen YE, Yang Q (2004). Cardiomyocyte-restricted peroxisome proliferator-activated receptor-delta deletion perturbs myocardial fatty acid oxidation and leads to cardiomyopathy. Nat Med.

[27] Heinonen SE, Merentie M, Hedman M, Makinen PI, Loponen E, Kholova I, Bosch F, Laakso M, Yla-Herttuala S (2011). Left ventricular dysfunction with reduced functional cardiac reserve in diabetic and non-diabetic LDL-receptor deficient apolipoprotein B100-only mice. Cardiovasc Diabetol.

[28] Zhao X, Yang H, Jiang G, Ni M, Deng Y, Cai J, Li Z, Shen F, Tao X (2014). Simvastatin attenuates radiation-induced tissue damage in mice. J Radiat Res.

[29] Ling MY, Ma ZY, Wang YY, Qi J, Liu L, Li L, Zhang Y (2013). Up-regulated ATP-sensitive potassium channels play a role in increased inflammation and plaque vulnerability in macrophages. Atherosclerosis.

[30] Ishimaru K, Ueno H, Kagitani S, Takabayashi D, Takata M, Inoue H (2007). Fasudil attenuates myocardial fibrosis in association with inhibition of monocyte/macrophage infiltration in the heart of DOCA/salt hypertensive rats. J Cardiovasc Pharmacol.

[31] Miao X, Wang Y, Sun J, Sun W, Tan Y, Cai L, Zheng Y, Su G, Liu Q, Wang Y (2013). Zinc protects against diabetes-induced pathogenic changes in the aorta: roles of metallothionein and nuclear factor (erythroid-derived 2)-like 2. Cardiovasc Diabetol.

[32] Da SJ, Santos-Silva MC, Cunha FQ, Assreuy J (2002). The role of ATP-sensitive potassium channels in neutrophil migration and plasma exudation. J Pharmacol Exp Ther.

[33] Weil BR, Manukyan MC, Herrmann JL, Wang Y, Abarbanell AM, Poynter JA, Meldrum DR (2010). Mesenchymal stem cells attenuate myocardial functional depression and reduce systemic and myocardial inflammation during endotoxemia. Surgery.

[34] Simon F, Fernandez R (2009). Early lipopolysaccharide-induced reactive oxygen species production evokes necrotic cell death in human umbilical vein endothelial cells. J Hypertens.

[35] Ting C, Bansal V, Batal I, Mounayar M, Chabtini L, El AG, Azzi J (2012). Impairment of immune systems in diabetes. Adv Exp Med Biol.

[36] Lukewich MK, Lomax AE (2014). Endotoxemia enhances catecholamine secretion from male mouse adrenal chromaffin cells through an increase in Ca (2+) release from the endoplasmic reticulum. Endocrinology.

[37] O'Sullivan L, Cuffe JS, Paravicini TM, Campbell S, Dickinson H, Singh RR, Gezmish O, Black MJ, Moritz KM (2013). Prenatal exposure to dexamethasone in the mouse alters cardiac growth patterns and increases pulse pressure in aged male offspring. PLoS One.

[38] Angus DC, Linde-Zwirble WT, Lidicker J, Clermont G, Carcillo J, Pinsky MR (2001). Epidemiology of severe sepsis in the United States: analysis of incidence, outcome, and associated costs of care. Crit Care Med.

[39] Chhabra P, Brayman KL (2013). Stem cell therapy to cure type 1 diabetes: from hype to hope. Stem Cells Transl Med.

[40] Grishman EK, White PC, Savani RC (2012). Toll-like receptors, the NLRP3 inflammasome, and interleukin-1beta in the development and progression of type 1 diabetes. Pediatr Res.

[41] Wen HL, Liang ZS, Zhang R, Yang K (2013). Anti-inflammatory effects of triptolide improve left ventricular function in a rat model of diabetic cardiomyopathy. Cardiovasc Diabetol.

[42] Bilim O, Takeishi Y, Kitahara T, Arimoto T, Niizeki T, Sasaki T, Goto K, Kubota I (2008). Diacylglycerol kinase zeta inhibits myocardial atrophy and restores cardiac dysfunction in streptozotocin-induced diabetes mellitus. Cardiovasc Diabetol.

[43] Regan TJ, Lyons MM, Ahmed SS, Levinson GE, Oldewurtel HA, Ahmad MR, Haider B (1977). Evidence for cardiomyopathy in familial diabetes mellitus. J Clin Invest.

[44] Nunes S, Soares E, Fernandes J, Viana S, Carvalho E, Pereira FC, Reis F (2013). Early cardiac changes in a rat model of prediabetes: brain natriuretic peptide overexpression seems to be the best marker. Cardiovasc Diabetol.

[45] Lu H, Raptis M, Black E, Stan M, Amar S, Graves DT (2004). Influence of diabetes on the exacerbation of an inflammatory response in cardiovascular tissue. Endocrinology.

[46] Becerra A, Echeverria C, Varela D, Sarmiento D, Armisen R, Nunez-Villena F, Montecinos M, Simon F (2011). Transient receptor potential melastatin 4 inhibition prevents lipopolysaccharide-induced endothelial cell death. Cardiovasc Res.

[47] Gerzanich V, Woo SK, Vennekens R, Tsymbalyuk O, Ivanova S, Ivanov A, Geng Z, Chen Z, Nilius B, Flockerzi V, Freichel M, Simard JM (2009). De novo expression of Trpm4 initiates secondary hemorrhage in spinal cord injury. Nat Med.

[48] Grand T, Demion M, Norez C, Mettey Y, Launay P, Becq F, Bois P, Guinamard R (2008). 9-phenanthrol inhibits human TRPM4 but not TRPM5 cationic channels. Br J Pharmacol.

[49] Simard C, Salle L, Rouet R, Guinamard R (2012). Transient receptor potential melastatin 4 inhibitor 9-phenanthrol abolishes arrhythmias induced by hypoxia and re-oxygenation in mouse ventricle. Br J Pharmacol.

[50] Simard JM, Geng Z, Woo SK, Ivanova S, Tosun C, Melnichenko L, Gerzanich V (2009). Glibenclamide reduces inflammation, vasogenic edema, and caspase-3 activation after subarachnoid hemorrhage. J Cereb Blood Flow Metab.

[51] Koh GC, Weehuizen TA, Breitbach K, Krause K, de Jong HK, Kager LM, Hoogendijk AJ, Bast A, Peacock SJ, van der Poll T, Steinmetz I, Wiersinga WJ (2013). Glyburide reduces bacterial dissemination in a mouse model of melioidosis. PLoS Negl Trop Dis.

