# Supplementary figures and images for: Glibenclamide attenuates myocardial injury by lipopolysaccharides in streptozotocin-induced diabetic mice
Source: Cardiovasc Diabetol. 2014 Jul 31;13:106. doi: 10.1186/s12933-014-0106-y (PMC4147163; doi:10.1186/s12933-014-0106-y)

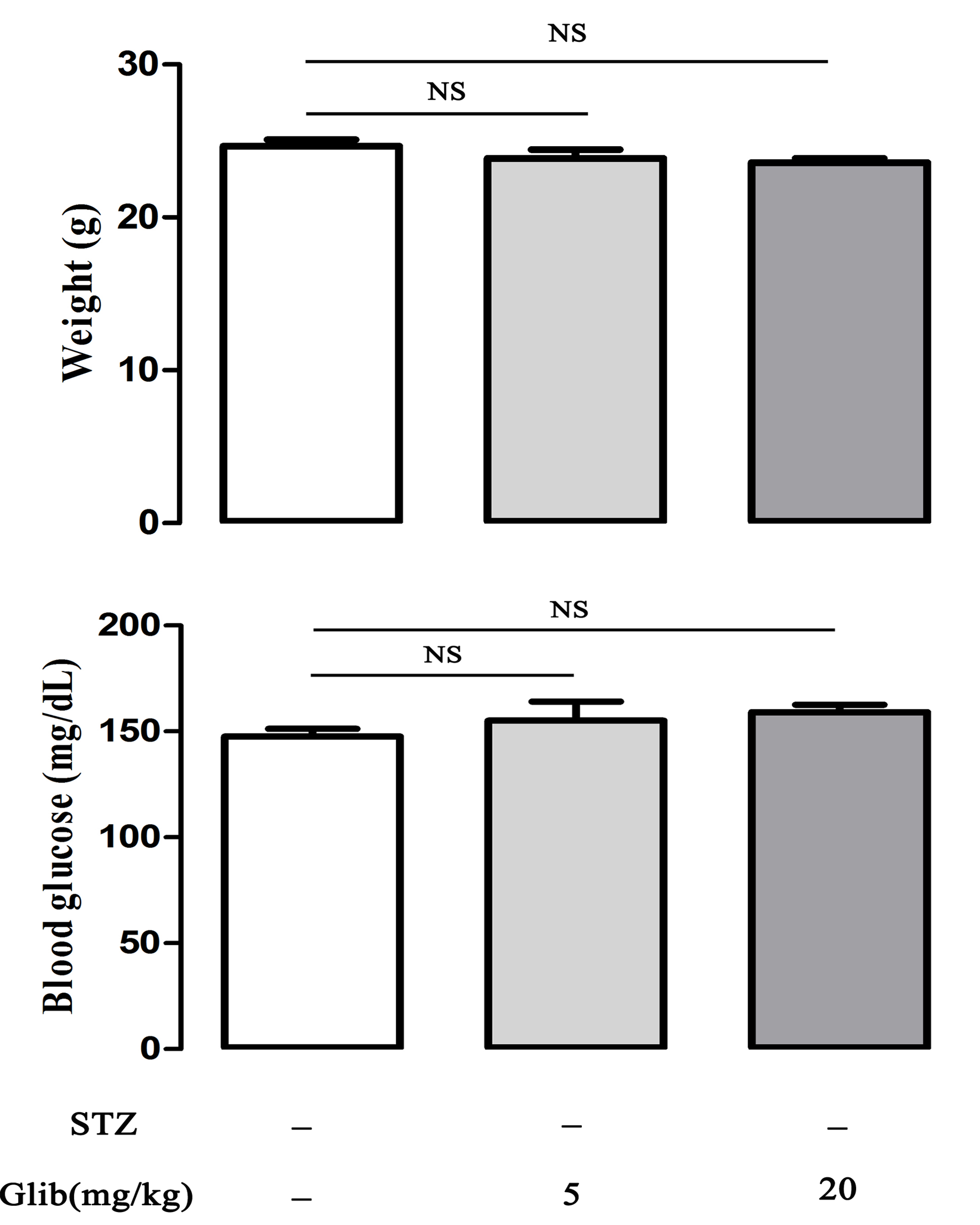

Supplement: Additional file 1: Figure S1. — Glibenclamide did not modify body weight and blood glucose in normal mice. Compared with control, glibenclamide (5 and 20 mg/kg, i.g, ×14 d) administration did not change body weight and blood glucose. NS means no significant difference. Values are means ± SEM (n = 5 per group). [file 12933_2014_106_MOESM1_ESM.tiff]

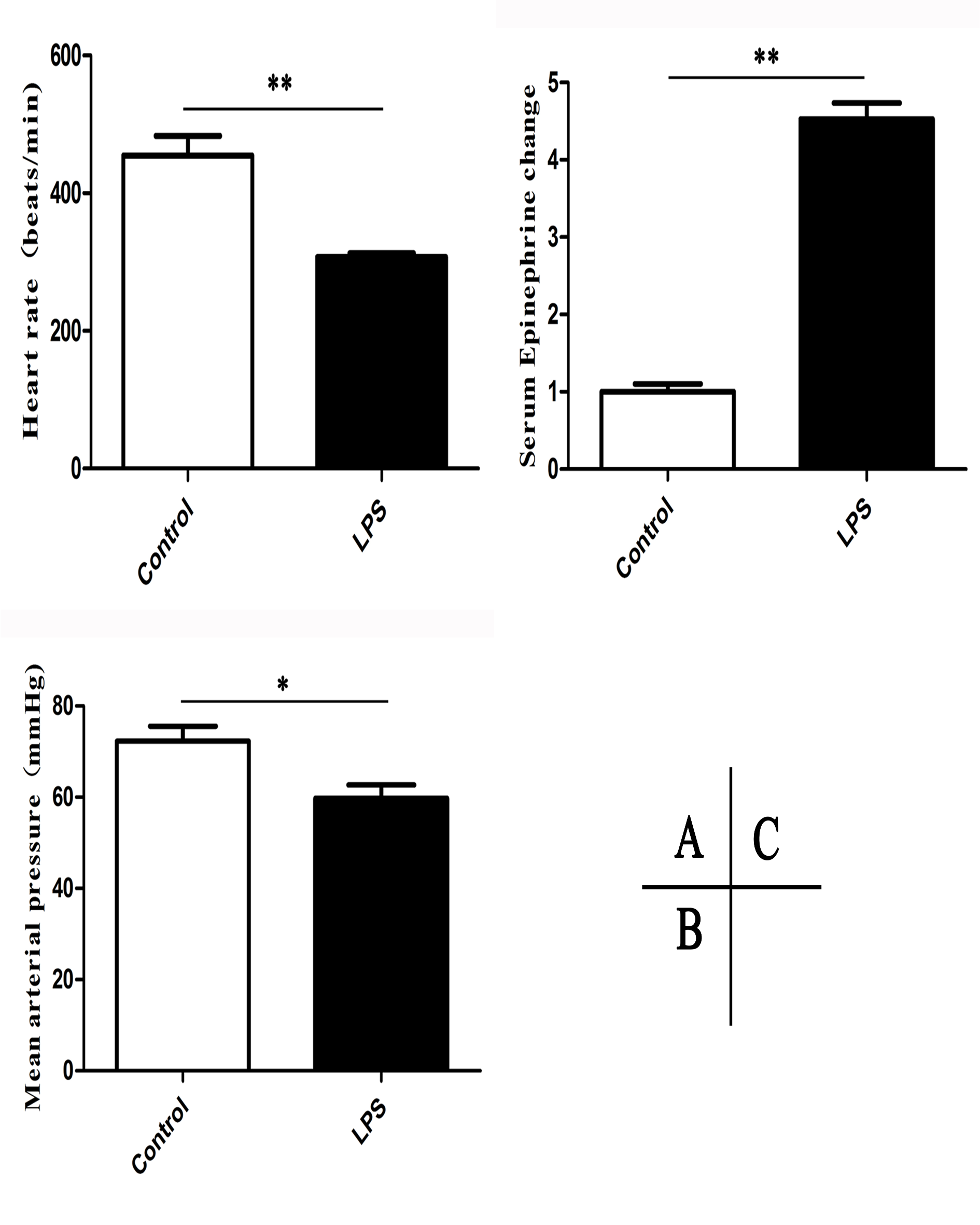

Supplement: Additional file 2: Figure S2. — Injection of LPS caused a decrease in heart rate and mean arterial pressure, and elevated serum epinephrine level in mice. Six hours after LPS (15 mg/kg) injection (i.p), heart rate (A) and mean arterial pressure (B) were measured by MPA-HBBS software. Serum epinephrine (C) level was assessed by ELISA. Mice were anesthetized with chloral hydrate (4%), heart rate and mean arterial pressure of endotoxemic mice decreased during LPS stimulation (heart rate and mean arterial pressure had been measured for 10 minutes). Serum epinephrine level was significantly increased in endotoxemic mice compared with control. *P < 0.05, **P < 0.01. Values are means ± SEM (n = 5 per group). [file 12933_2014_106_MOESM2_ESM.tiff]

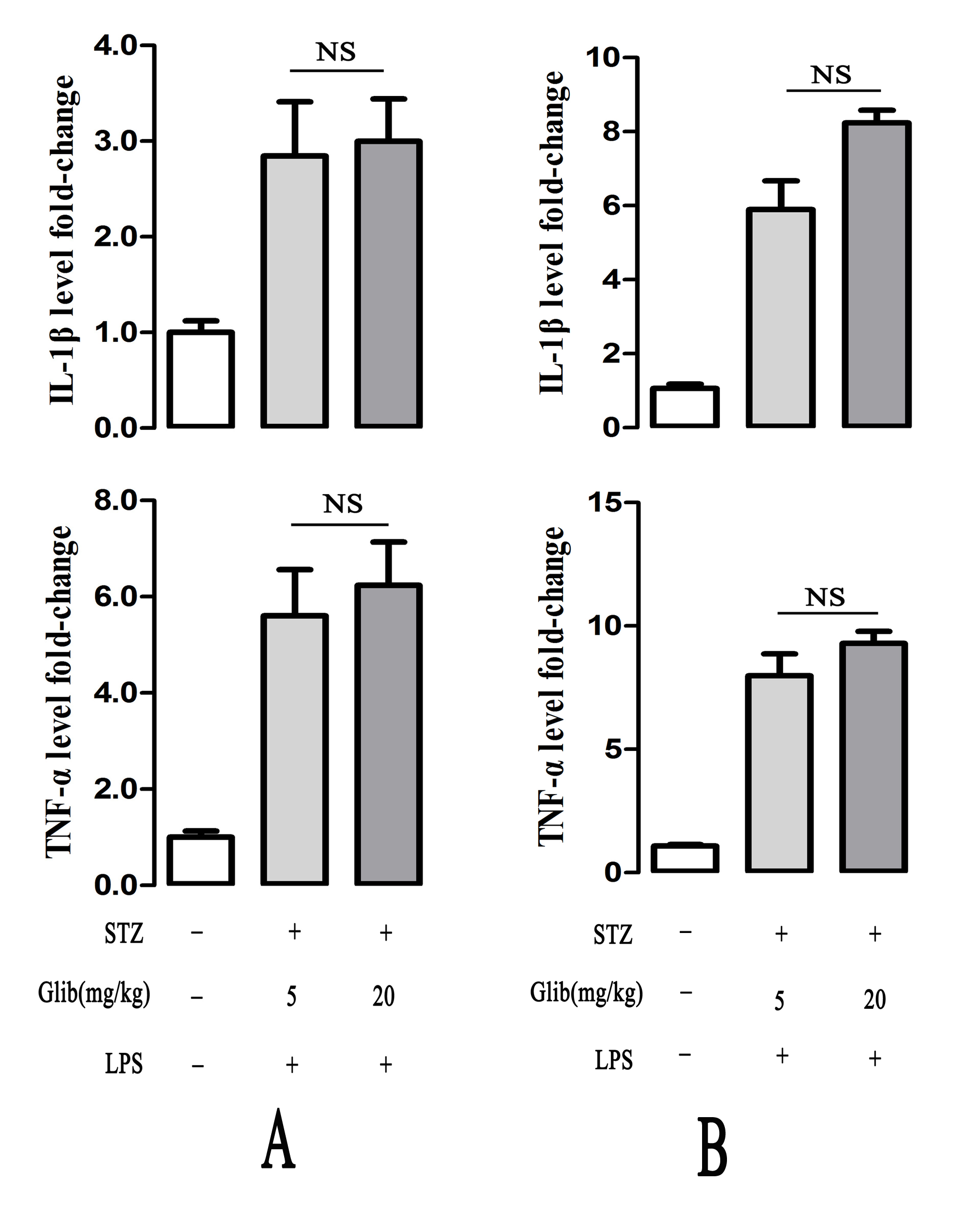

Supplement: Additional file 3: Figure S3. — IL-β and TNF-α levels in cardiac tissue and serum had no significant difference between LPS + STZ + Glibenclamide (5 mg/kg) group and LPS + STZ + Glibenclamide (20 mg/kg) group. IL-1β and TNF-α levels in cardiac tissue and serum were assessed by ELISA 6 h after LPS injection (15 mg/kg, i.p). A: Cardiac tissue, B: Serum. NS means no significant difference. Values are means ± SEM (n = 6-8 per group). [file 12933_2014_106_MOESM3_ESM.tiff]
